# Supplementary material for: Phosphoinositide 3-Kinase (PI3K) Reactive Oxygen Species (ROS)-Activated Prodrug in Combination with Anthracycline Impairs PI3K Signaling, Increases DNA Damage Response and Reduces Breast Cancer Cell Growth
Source: Int J Mol Sci. 2021 Feb 19;22(4):2088. doi: 10.3390/ijms22042088 (PMC7923228; doi:10.3390/ijms22042088)
Supplement: Supplementary file 1 [file ijms-22-02088-s001.pdf]

Table S1. Comparison of IC50 values of RIDR-PI-103 vs PI-103.

Cell lines	IC50 RIDR-PI-103 (0–110 µM)	IC50 PI-103 (0–5 µM)
Fibroblast	N.D.	3.9 µM
MCF10A	78.6 µM	3.8 µM
MDA-MB-231	47.3 µM	N.D.
MDA-MB-361	43.0 µM	1.7 µM
MDA-MB-453	49.1 µM	0.82 µM

Table S2. IC50 values of DOXO, DOXO ± 10 µM RIDR, DOXO ± 15 µM RIDR and DOXO ± 30 µM RIDR in MDA-MB-231, MDA-MB-361 and MDA-MB-453 cell lines.

Treatment group	IC50 (MDA-MB-231)	IC50 (MDA-MB-361)	IC50 (MDA-MB-453)
Doxorubicin	405.3 nM	116 nM	578.5 nM
DOXO +10 µM RIDR-PI-103	184.9 nM	111 nM	529.5 nM
DOXO +15 µM RIDR-PI-103	146.5 nM	109.7 nM	516 nM
DOXO + 30 µM RIDR-PI-103	N.D.	73.2 nM	477.8 nM

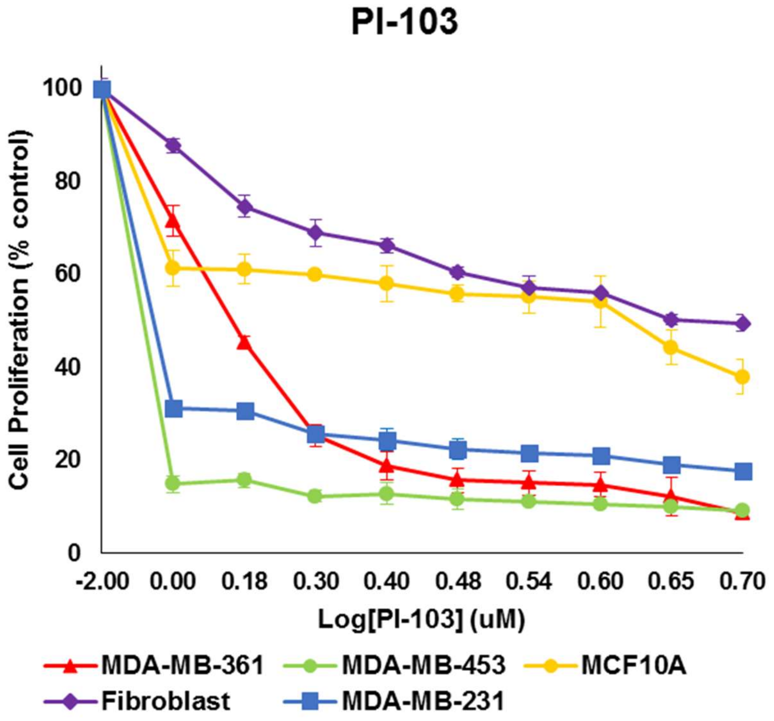

Figure 1. Dose response of PI-103 in MCF10A, normal fibroblasts and breast cancer cells. NH fibroblasts, MCF10A, MDA-MB-231, MDA-MB-361 and MDA-MB-453 cells were plated in triplicate in 96 well plates and treated with PI-103 (0-5 μM) for 72 hour. The data was analyzed and presented using GraphPad Prism 7 (n=3 independent experiments performed in triplicate ± SEM). IC50 values were as follows: Fibroblast: 3.9 μM, MCF10A: 3.8 μM, MDA-MB-231: N.D., MDA-MB-453: 0.82 μM, MDA-MB-361: 1.7 μM.

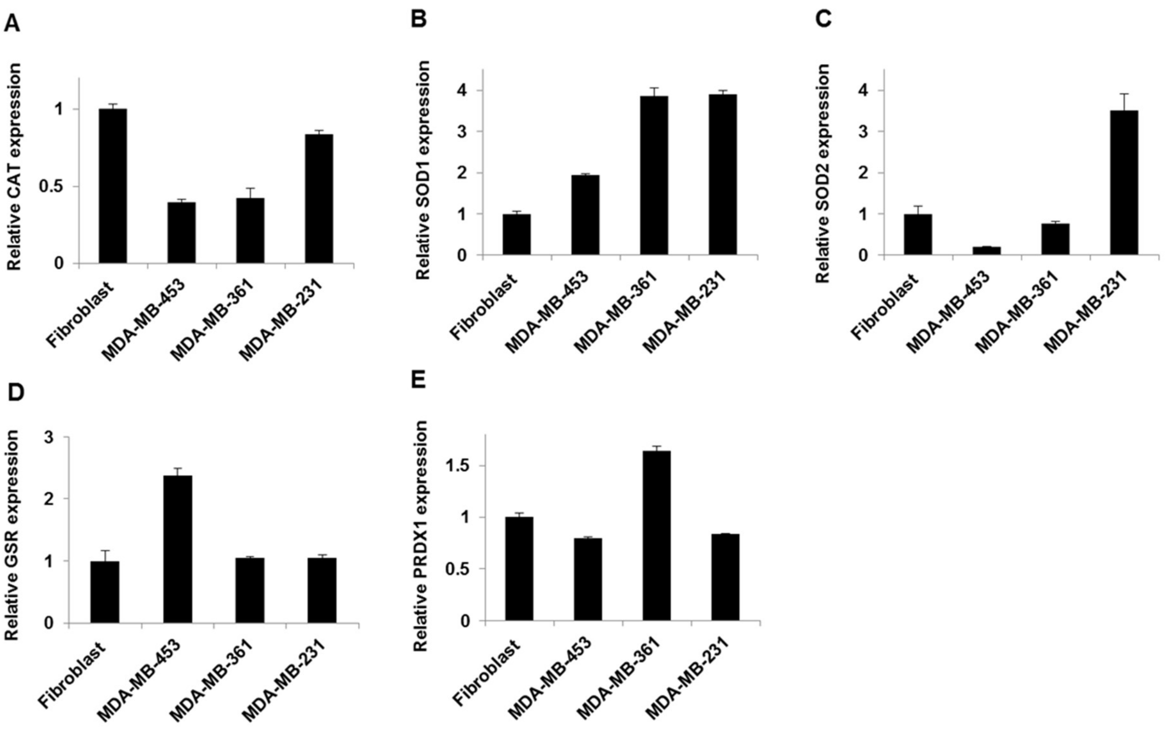

Figure S2: Endogenous antioxidant levels in fibroblast and breast cancer cells. Equal number of NH fibroblasts, MDA-MB-231, MDA-MB-361 and MDA-MB-453 cells was plated. Total RNA was extracted from these cell lines using the RNA extraction mini according to the manufacturer's instruction. Two-step Real Time-qPCR was performed to assess the mRNA level of Catalase, SOD1, SOD2, PRDX1 and GSR. First strand cDNA was synthesized. qPCR was performed using CFX96 Real-Time System. Actin was used as an internal control. Relative Catalase, SOD1, SOD2, PRDX1 and GSR mRNA expression was presented by $2^{-\Delta\Delta CT}$ method.

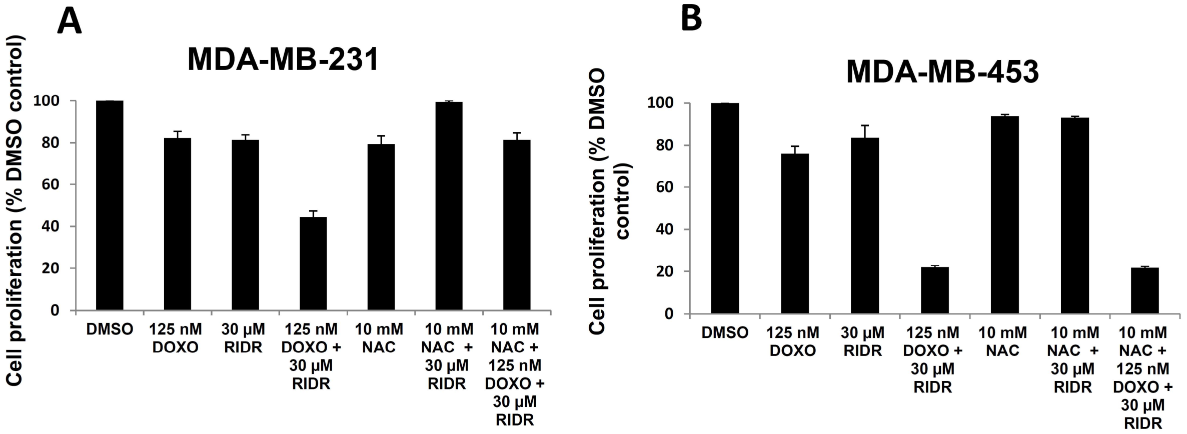

Figure S3: NAC, a ROS scavenger rescues the antiproliferative effects of combination RIDR-PI-103 and doxorubicin in MDA-MB-231 but not MDA-MB-453 cells. (A) MDA-MB-231 ($2x10^4$cells/well) were seeded in 96 well plates in triplicate and treated with vehicle (DMSO), 125 nM Doxorubicin (DOXO) ± 30 µM RIDR-PI-103 (RIDR) and 10 mM N-acetyl-cysteine (NAC) for 24 hour. Cells were treated with MTT (5 mg/ml) for 4 hour and absorbance read at 570 nm in a microtiter plate reader (n=2 independent experiments performed in triplicate ± SEM). (B) MDA-MB-453 ($2x10^4$cells/well) were seeded in 96 well plates in triplicate and treated with vehicle (DMSO), 125 nM Doxorubicin (DOXO) ± 30 µM RIDR-PI-103 (RIDR) and 10 mM N-acetyl-cysteine (NAC) for 72 hour. Cells were treated with MTT (5 mg/ml) for 4 hour and absorbance read at 570 nm in a microtiter plate reader (n=2 independent experiments performed in triplicate ± SEM).

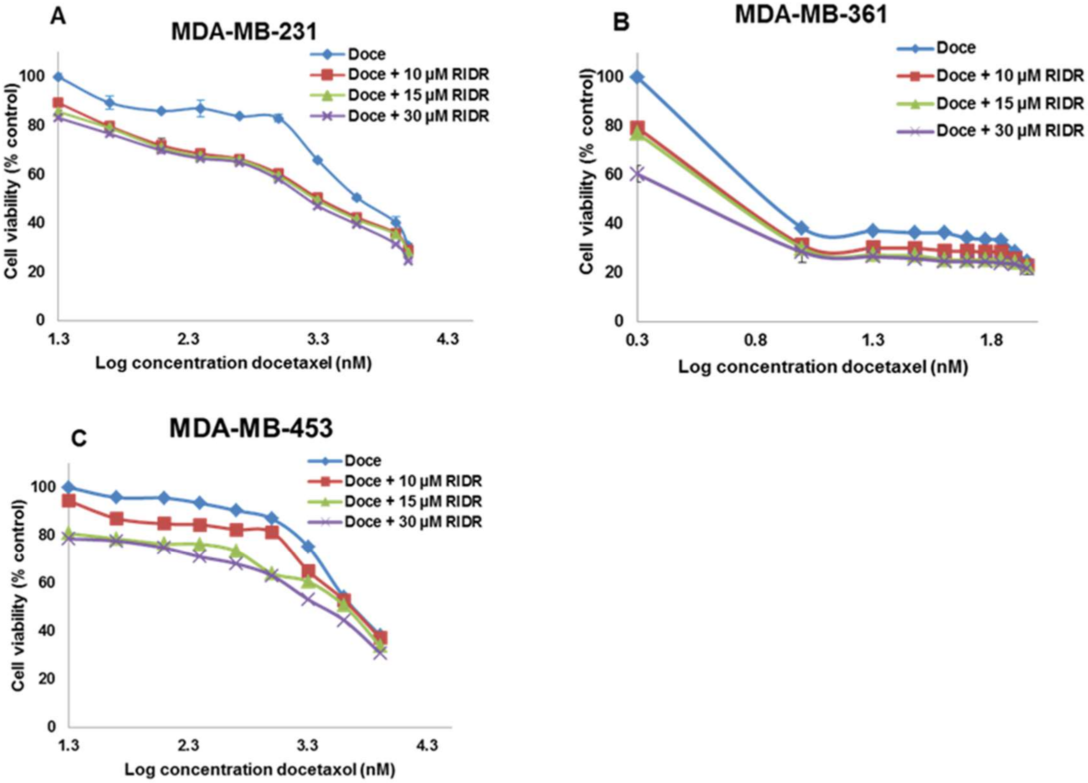

Figure S4: Docetaxel did not sensitize RIDR-PI-103 in breast cancer cells. (A-C) MDA-MB-231, MDA-MB-361 and MDA-MB-453 cells (2x10^{4}cells/well) were seeded in 96 well plates in triplicate and treated with a serial dose of docetaxel in presence of 10 µM, 15 µM and 30 µM RIDR-PI-103 for 72 hour. The concentration of docetaxel used was 50-50000 pM docetaxel for MDA-MB-231 cells, 0-90 nM for MDA-MB-361 cells and 50-10000 pM for MDA-MB-453 cells. Cells were treated with MTT (5 mg/ml) for 4 hour and absorbance read at 570 nm in a microtiter plate reader.

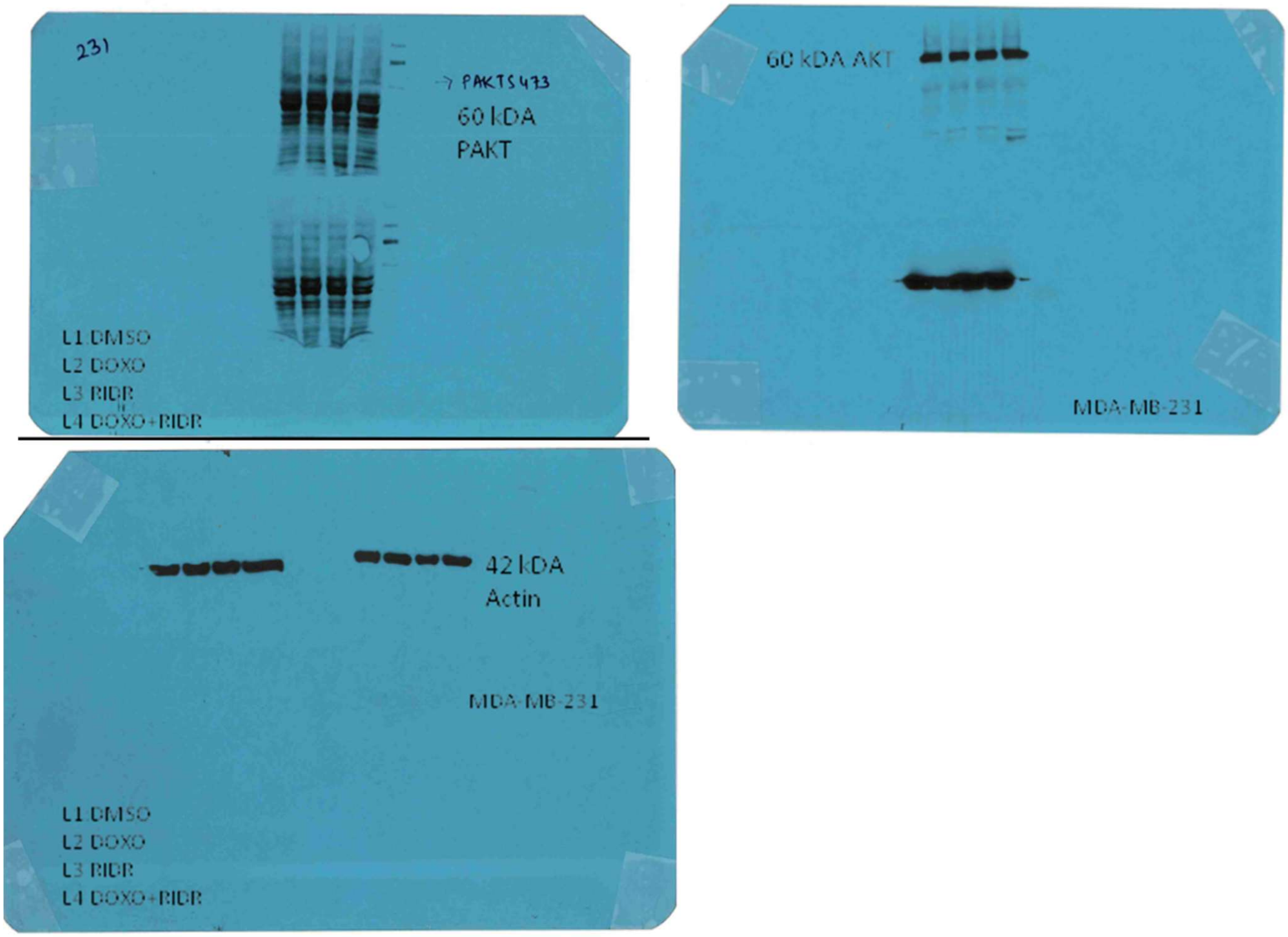

Figure S5: Whole blot image of p-AKT/AKT/Actin in MDA-MB-231 cells from Figure 6A.

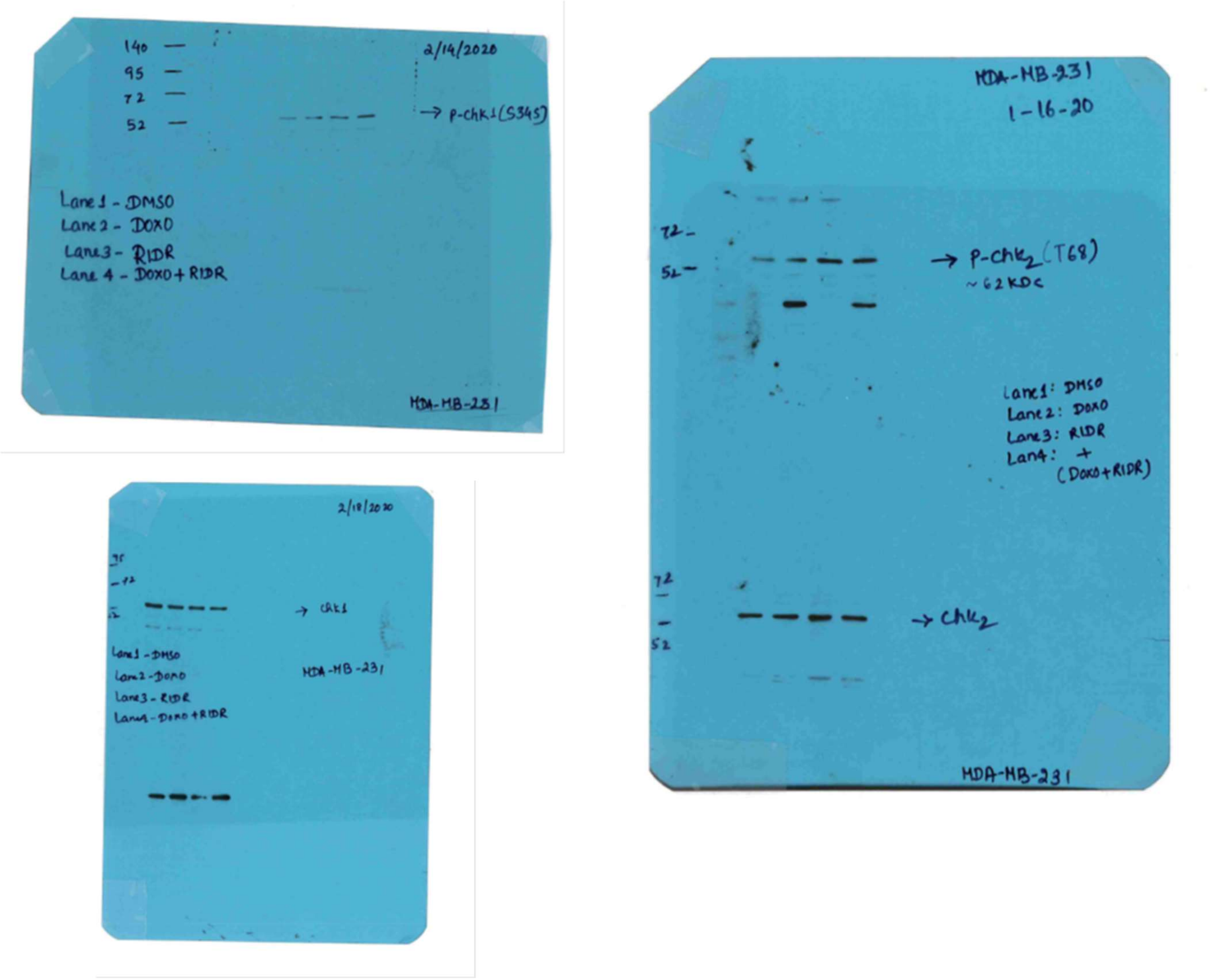

Figure S6. Whole blot image of p-CHK1S345/CHK1 and p-CHK2T68/CHK2 in MDA-MB-231 cells from Figure 6A.

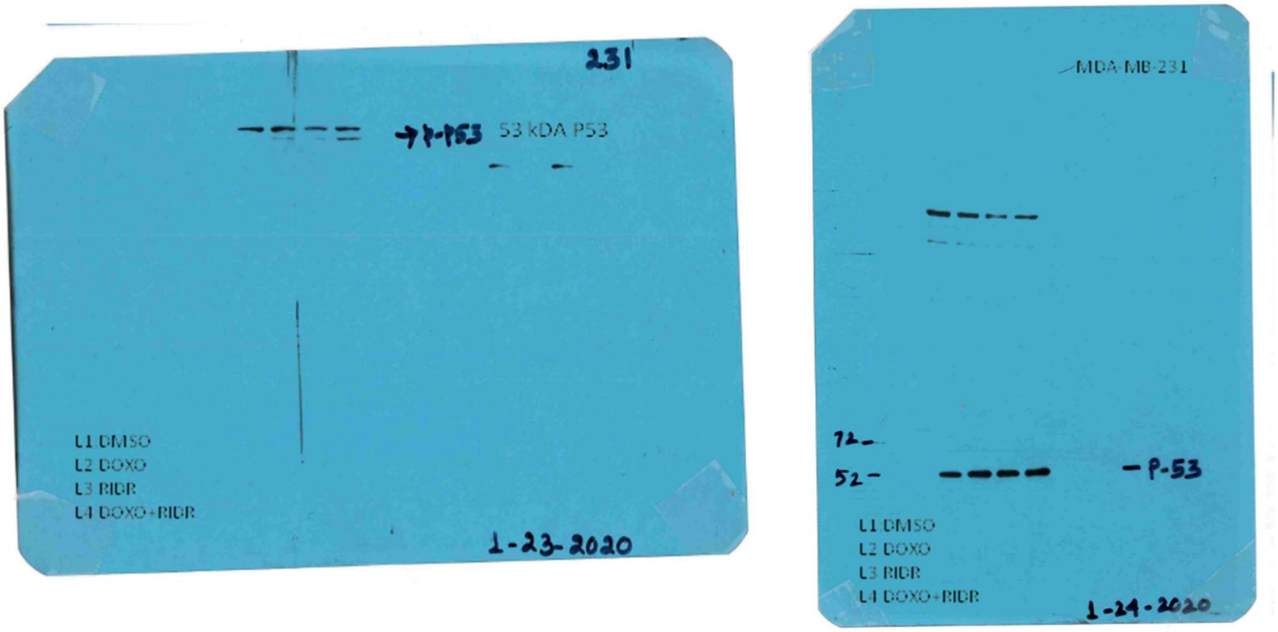

Figure S7. Whole blot image of p-p53 and p53 in MDA-MB-231 cells from Figure 6A.

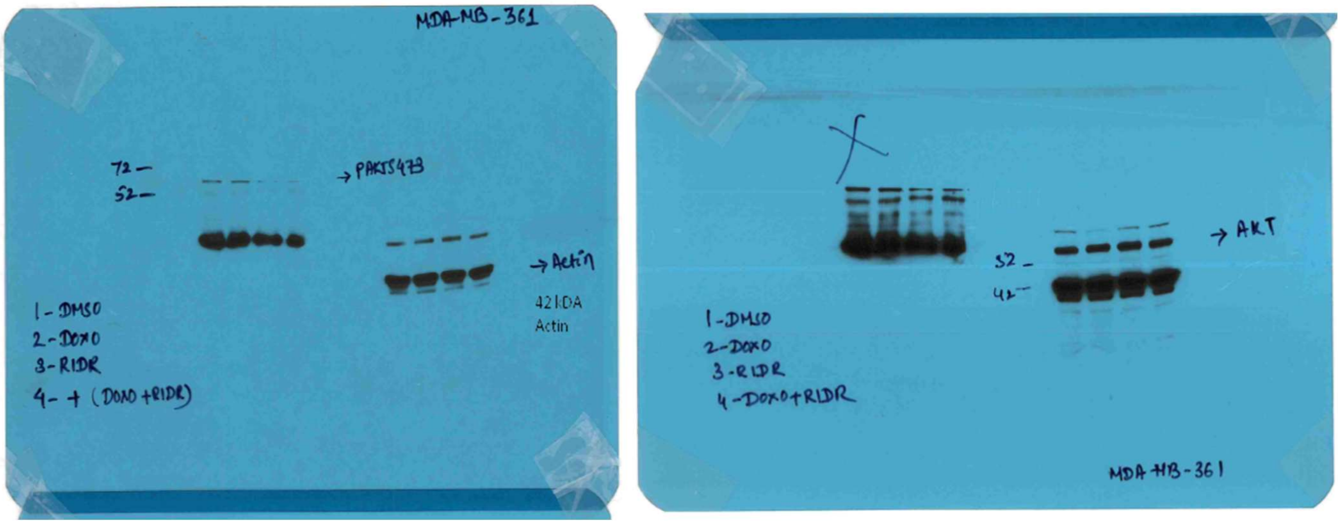

Figure S8: Whole blot image of p-AKT/AKT/Actin in MDA-MB-361 cells from Figure 6B.

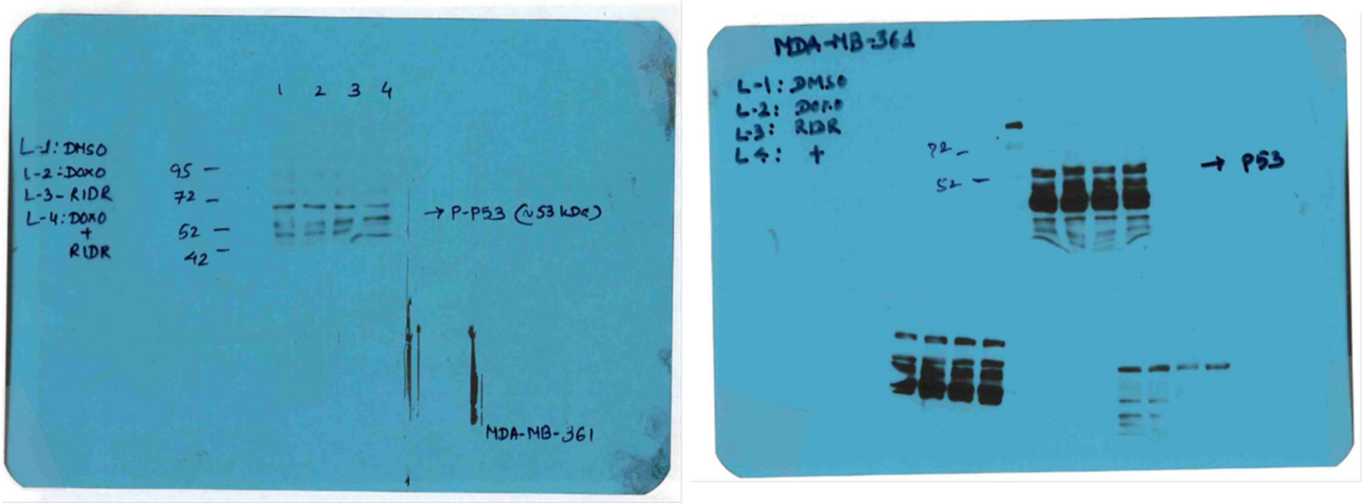

Figure S9. Whole blot image of p-p53 and p53 in MDA-MB-361 cells from Figure 6B.

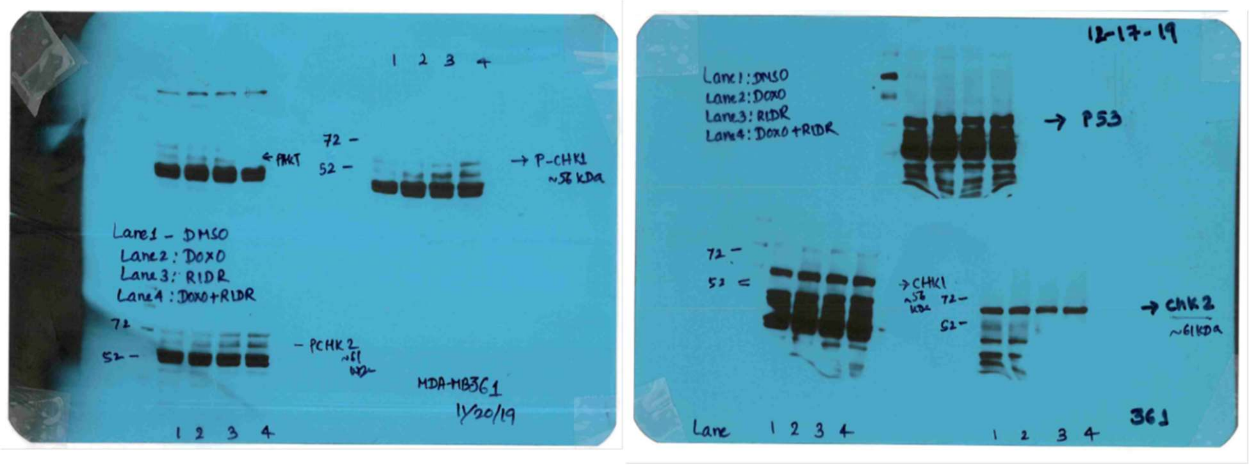

Figure S10. Whole blot image of p-CHK1S345/CHK1 and p-CHK2T68/CHK2 in MDA-MB-361 cells from Figure 6B.

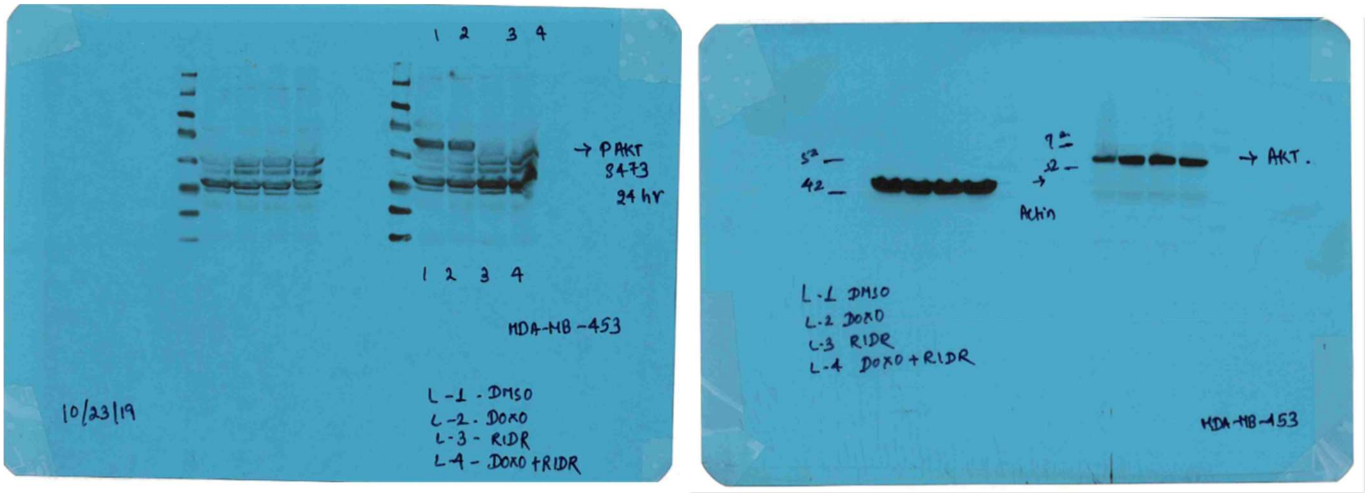

Figure S11. Whole blot image of p-AKT/AKT/Actin in MDA-MB-453 cells from Figure 6C.

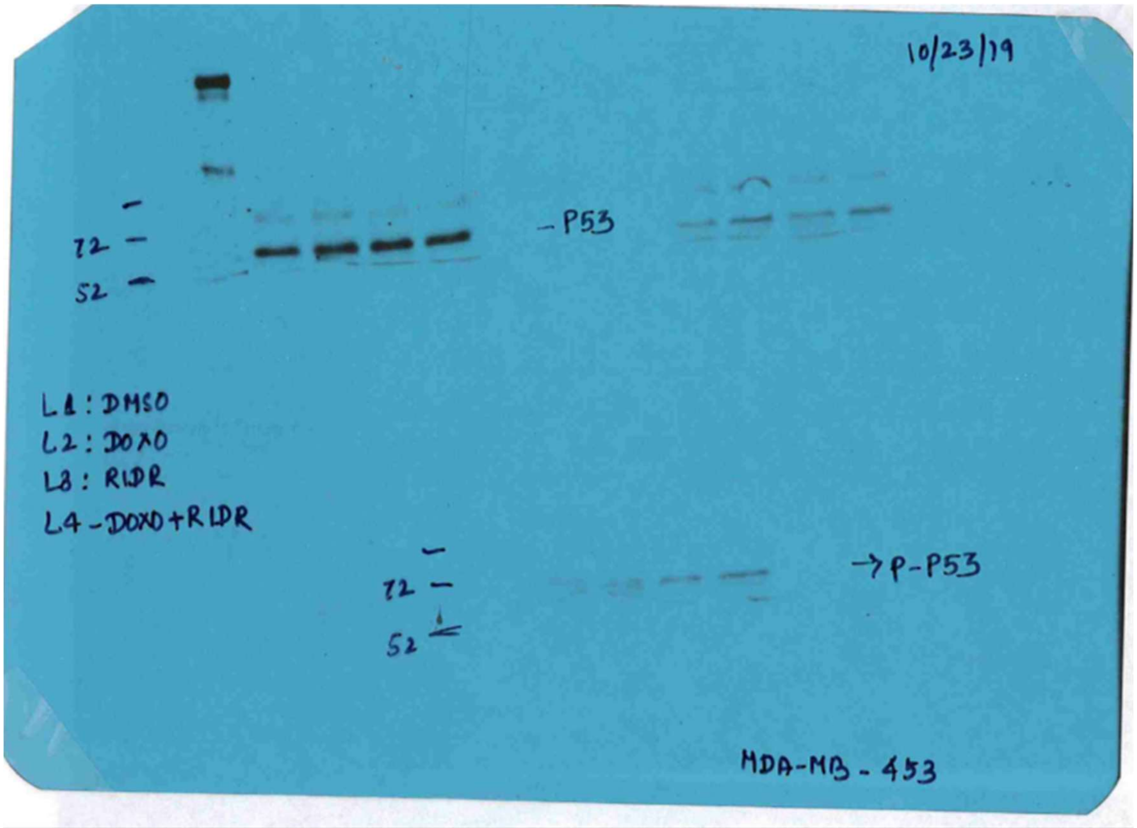

Figure S12. Whole blot image of p-p53 and p53 in MDA-MB-453 cells from Figure 6C.

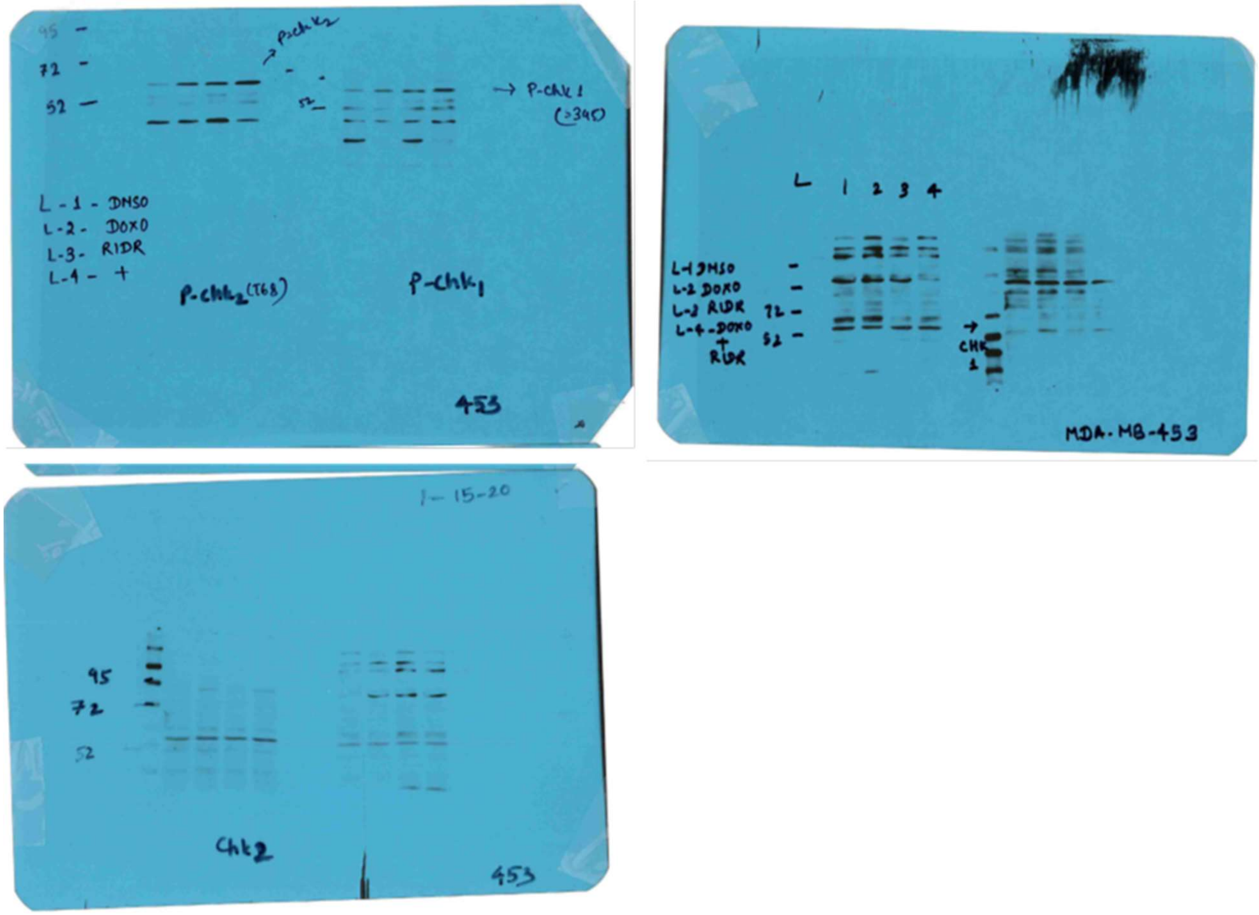

Figure S13. Whole blot image of p-CHK1S345/CHK1 and p CHK2T68/CHK2 in MDA-MB-453 cells from Figure 6C.
